# The relationship between perception of control and mood: The intervening effect of cultural values in a Saudi Arabian sample

**DOI:** 10.1371/journal.pone.0220509

**Published:** 2019-08-22

**Authors:** Salha Senan, Rachel. M. Msetfi, Mogeda El Keshky, Yemaya Halbrook

**Affiliations:** 1 Department of Psychology, King Abdulaziz University, Jeddah, Saudi Arabia; 2 Centre for Social Issues Research, Department of Psychology, University of Limerick, Limerick, Republic of Ireland; 3 Health Research Institute, University of Limerick, Limerick, Republic of Ireland; 4 Computational Psychopathology Research Group, University of Oxford, Oxford, United Kingdom; 5 Department of Psychology, Faculty of Arts, Assiut University, Assiut, Egypt; Universidad de Granada, SPAIN

## Abstract

The relationship between the constructs of perceived control and symptoms of mood disorders has been demonstrated. The current study evaluates cultural values both as an individual difference moderating variable and as one of the mechanisms through which the association between perceived control and mood disturbances may operate. The hypotheses were examined with a sample of 615 participants recruited in Saudi Arabia. Participants completed measures of perceived control, individualism and collectivism, and symptoms of depression and bipolar disorder. In general, the results supported a model in which higher levels of perceived control promote a less symptomatic mood state. In most cases, cultural values positively mediated the relationship between perceived control and mood disturbance with lower symptom levels predicted. However, when the components of perceived control were examined separately, high perceived mastery together with highly individualistic values predicted higher levels of bipolar symptoms. In this sample, there was less evidence of cultural values moderating the control–mood disturbance relationship. Only one moderator relationship was identified, which showed low control linking to higher symptom levels only in those who disagreed with individualistic values. Overall, our data are in agreement with the notion that pre-existing cultural values have an important effect on mood disorder symptoms.

## Introduction

When people feel that they have control over their lives, they also tend to be healthier than people who feel that they lack control [1]. For example, people with strong perceptions of control have been reported to experience better outcomes in relation to diseases, such as cancer [2], chronic illnesses like diabetes [3] and heart disease [4], as well as improved treatment adherence and effectiveness [5]. On the other hand, feeling helpless and perceiving little control over one’s life has been linked to poorer health [6], higher mortality rates [7] and the presence of mood disorders like depression [8], as well as severe symptoms like helplessness and suicidal feelings [9]. So, it seems that a strong perception of control is a protective factor in relation to physical and mental healthiness. In the current study, we focus particularly on mood states and we question whether the relationship between perceived control and health is as simple as suggested above. As we describe below, we aim to test whether the values a person holds influence, either to enhance or to diminish, the effects of this protective factor, especially in relation to the symptoms of specific types of mood disorders. First, however, we provide a brief introduction to the links between perceived control and healthiness before discussing a values-based causal pathway hypothesis.

### Control and healthiness

Studies conducted over many years have evidenced the strong links between perceived control and health states (see [10] for an up to date account). In recent years, there have also been attempts to manipulate and enhance perceived control experimentally [11, 12]. For example, in experiments, instructions that influence the nature of information sampled in a given situation can increase perceptions of control [13, 14]. One instance of this is that increasing levels of behaviour via instruction enables participants to experience high levels of action-outcome occurrences. As a consequence, the contingency between participant behaviour and outcomes increases and thus their perceptions of control increases also [14]. Another experimental example is that increasing attention to control enhancing information, again via instructions, can also increase perceived control [11, 12]. These findings indicate that perceptions of control are amenable to change. Indeed, some recommendations suggest that perceived control may be enhanced to improve wellbeing as part of psychological therapies [10].

The overall implication of this is that higher levels of perceived control are a universally healthy characteristic. However, this is not always the case. Symptom levels have been shown to be related to unhealthy or maladaptive increases in control. For example, high perceived control is sometimes thought of as ‘illusory control’, which is defined as ‘overestimating the influence that one’s actions have over uncontrollable events’ [15] and is linked to beliefs about the effectiveness of bogus treatments for diseases [16], and excessive risk taking [17]. Indeed, individuals with schizophrenia have been shown to be more susceptible to illusory control than others [18, 19], and higher levels of obsessive compulsive disorder symptoms are correlated with higher levels of perceived control [20]. Thus, high levels of perceived control may not always be an index of healthiness and this is dependent on the type of symptom implicated.

### Control, culture, and healthiness

Another important question is whether a goal of improving perceived control is appropriate to people in all populations, and whether people’s values influence the pathway between perceived control and symptomatology. Values that are related to the extent to which a person views the world through individualistic and collectivistic lenses affect the importance that an individual may place on feelings of control [21]. Consistent with this, data from the World Values Survey taken from 33 countries showed that whilst relationships between control and wellbeing were present in Asian samples, usually thought of as endorsing more collectivist values and placing less value on control, they were weaker than for non-Asian samples [22]. However, Steptoe et al., [23] also looked for differences in their data collected from 23 countries, and found that the strength of the relation between perceived control and depression did not vary across countries.

The relation between cultural values varies, as one might expect, depending on the type of mood disorder symptom studied [24]. For example, Msetfi et al., [24] carried out a study within a European population (UK and Ireland), and reported that the cultural values of individualism and collectivism mediated the relationship between experimentally manipulated perceived control derived from a behavioural task and mood disorder symptoms. Higher levels of collectivism were a protective factor in relation to low perceived control and depression but only in more diverse samples. However, high levels of perceived control and high levels of individualism were linked to increased euphoric bipolar symptoms. Based on this evidence, the question is whether similar patterns will be evident in populations thought to place less value on perceived control.

**Fig 1:**
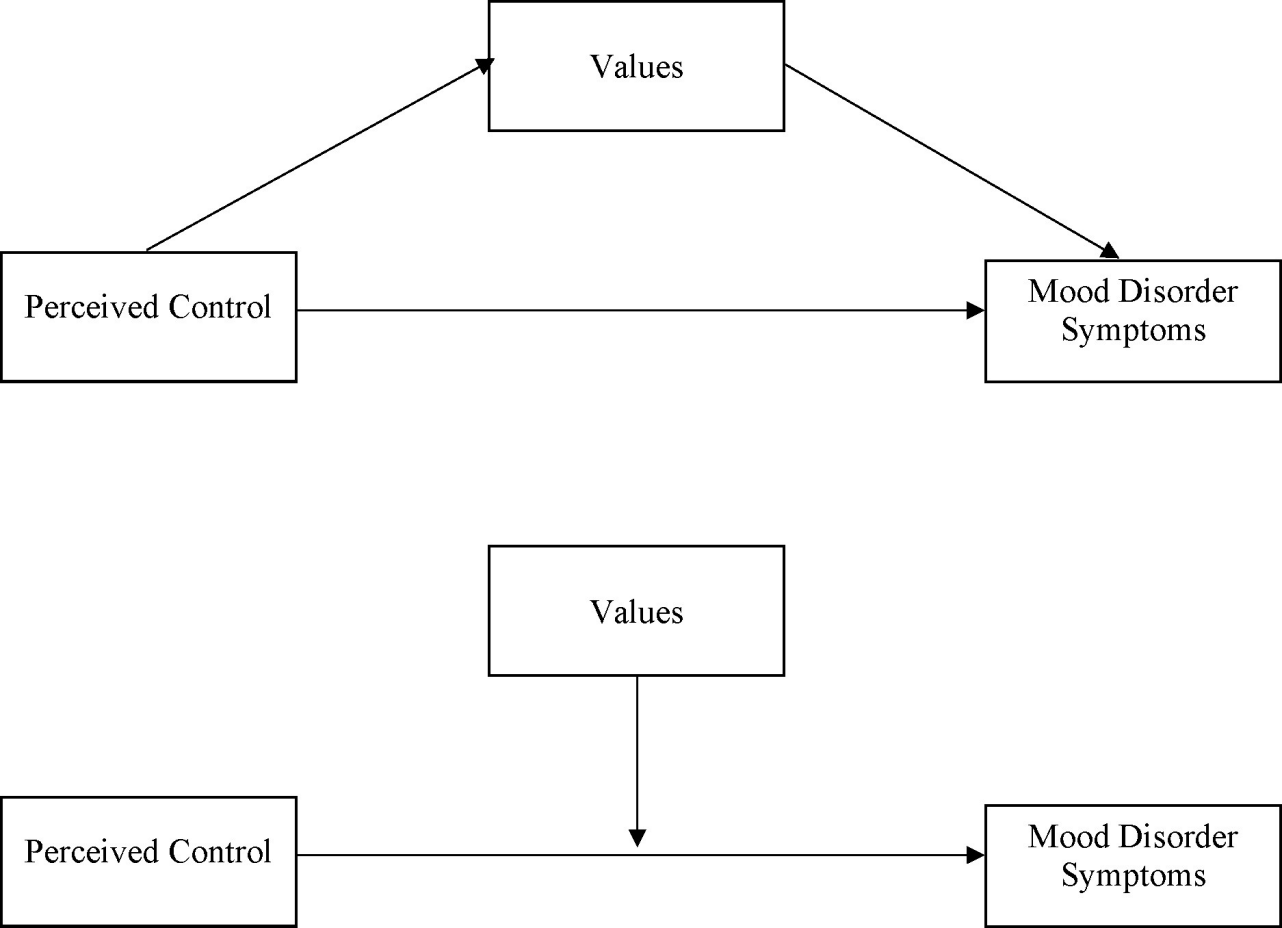
**The conceptual Perceived Control Mental Health Model (PCMHM) with mood disorder symptoms as the outcome variable and cultural values as the mediator variable (top) and a moderator variable (bottom)**.

The current study builds on the previous body of work. Here we study perceived control and mood disorder symptoms within a sample that we assume to endorse collectivist values relatively strongly. We also assume that levels of perceived control influence mood disorder symptoms in a manner dependent on the extent to which people endorse such cultural values and the importance they place on control. However, we note that previous work has tested different versions of this model (see Fig 1). The experimental study described above, which used perceived control measures derived from a behavioural task, tested a mediator hypothesis (see Fig 1 top). In contrast, the cross cultural observational studies described above tested, what are essentially, moderator hypotheses; that cultural values influence the strength and / or direction of a relationship (see Fig 1 bottom). In the current study, we aim to test both sets of hypotheses and will address the theoretical and conceptual implications of the two models in the discussion.

### Current study

There are a number of methods of measuring and conceptualising perceived control [25]. Some previous work in this field has used objective measures of perception of control [24]. Objective measures refer to experiments in which participants assign numeric ratings to their experiences of specific contingencies involving their own actions and outcomes [26]. This type of work speaks to the accuracy with which people can assess the control they ‘actually’ have in a specific situation or situations. Another way of measuring perceived control is to ask people about their general beliefs about their own sense of control, in a manner which transcends specific situations. Lachman and Weaver [27] defined the general sense of control as being composed of two aspects. These are perceived mastery, which refers to a person’s perception of the effectiveness of their actions, and perceived constraints, which are constraints that interfere with their control. Skinner [25] argues that these are the essential components to perceived control and map on to the notion of ‘competence’ [am I effective] and ‘contingency’ [i.e. are my actions effective] respectively. Interference with either component of perceived control would be predicted to result in loss of control. In this study, we focussed on the general sense of control as a learned and generalised belief, which is predicted to impact symptoms.

As well as testing these vital constructs of control, we also planned to test a large sample of volunteers recruited from Saudi Arabia, a population generally considered to place less importance on perceived control and endorse more collectivist values. Previous work showed the relationship between control and depression to be similar across 23 countries [23] although anxiety was found to be weaker in collectivist samples [28]. Here, we chose to measure two types of mood disorder symptoms, including the symptoms of depression and the bipolar disorder, which have yielded different patterns of findings in relation to perceived control in our previous work [24]. All measures required for the study were currently available translated into Arabic with the exception of the Perception of Control questionnaire. Therefore, we first conducted a pilot study to translate, adapt and validate this measure for use with an Arabic population.

## Pilot study

The English version of the Sense of Control Measure was adapted and translated using the back-translation method procedures [29]. The scale was translated into Arabic by two of the authors (SS and AH), and then back-translated in to English by a professor from the English Centre of King Abdulaziz University who specialises in English linguistics. Then, both authors and the English language professor checked the final version of the scale in order to ensure that they were similar to the original English versions. The final version of the scale was then administered to 10 university students to test its clarity. No items were significantly changed during the process of translation and testing.

In order to evaluate the internal consistency of the scales, a total of 170 Saudi participants completed the scale (female *n* = 138, male *n* = 32). Their mean age was 26.33 years (*SD = 12*.*29*). Internal consistency was evaluated using Cronbach's alpha coefficients, with alpha values for the sense of control total and subscale scores being acceptable (all > .6; total, α = 0.73; perceived constraints, α = 0.76; perceived mastery, α = 0.61 respectively). The Arabic version of the sense of control scale was therefore considered appropriate and valid for use in the main study.

## Main study

## Materials and methods

### Participants

In total, 614 participants recruited from a population in Saudi Arabia took part in this study (female *n* = 510, male n = 104). The majority (69%) of participants were aged between 18 and 35 years (*n* = 298, 18–24, n = 126, 24–35), 90 reported being 35–44 years, 78 reported being 45–54, 18 reported being 55–64, and 4 reported being 65–74. All but two participants reported being Muslim and only 1.8% self-reported non-Arabic in nationality.

Participants’ average scores for perceived control were at the lower end of the ‘have control’ range, (perceived constraint: *M* = 34.45, *SD* = 8.80, where an ambivalent score (in the range of neither agree nor disagree with the statement) = 32; perceived mastery *M* = 22.50, *SD* = 4.24, ambivalent score = 16; perceived control *M* = 56.95, *SD* = 10.24, ambivalent score = 48). Additionally, the average BDI score was 11.88 (*SD* = 9.48, with 27.7% of participants scoring ≥ 16) and MDQ was 5.11 (*SD* = 3.13, with 30.1% of participants scoring ≥ 7) with cultural values scores as follows: horizontal individualism (*M* = 23.82, *SD* = 5.20), vertical individualism (*M* = 18.65, *SD* = 5.15), horizontal collectivism (*M* = 22.39, *SD* = 4.95), and vertical collectivism (*M* = 23.86, *SD* = 4.59).

### Measures

We already had access to some measures that were translated into Arabic. These are signalled using an asterisk* below (some were translated as part of one of the investigator’s PhD research [30]). Thus, only the measure of perception of control required translation as reported in the pilot study.

#### *Beck Depression Inventory [31]

The BDI is a self-report measure of depressed mood which has been used extensively with both clinical and student populations. This is a 21-item measure in which participants were asked to choose statements that best described them. The items ranged from neutral statements (e.g. I do not feel like a failure) scored as 0, to more extreme mood related statements (e.g. I feel I am a complete failure as a person) scored as up to a value of 3. Total scores could range from 0 to 63 with higher scores indicating higher levels of depression and a score of 16 or above indicating moderate levels of depression. The BDI has also been validated in college samples, correlations of .77 being reported between BDI scores and a psychiatric rating of severity of depression [32]. The BDI has been translated and validated in Arabic [33] and it is this version that is used here. In this study, Cronbach’s alpha gave .887, showing the scale has high reliability.

#### *Symptoms of Bipolar Disorder

The Mood Disorder Questionnaire [34] or MDQ is a self-report questionnaire designed to be used as a screening tool for bipolar disorder. Participants are asked, “Has there ever been a period of time when you were not your usual self and …” The statement is followed by 13 items related to symptoms of mania that can be answered yes or no, with a yes answer yielding a score of 1 (e.g., …you felt much more self-confident than usual?). Subsequent questions then enquire about the frequency and significance of the consequences of the items mentioned previously. In the present study, only the initial 13 items were scored and summed to produce a total score. This technique had been used in a number of studies that used a general population sample [35]. A score of 7 or more provides good sensitivity in terms of a bipolar disorder diagnosis, however here continuous scores were used rather than categorical groupings. This questionnaire was translated and validated in the Arabic by collaborators of this research group. Cronbach’s alpha for the current study was .761, showing that reliability was moderately high.

#### Cultural values

The Individualism and Collectivism scale IC [36] originally consisted of 32 self-report items that quantify the endorsement of distinct attributes of individualism and collectivism, known as horizontal and vertical dimensions. There are four subscales—Horizontal Individualism (HI), Vertical Individualism (VI), Horizontal Collectivism (HC), and Vertical Collectivism (VC), where horizontal dimensions emphasize equality and vertical subscales emphasize hierarchy. Thus, people with a more prominent HI pattern value self-reliance and uniqueness, and distinction from groups, whereas those for whom VI is more prominent are more likely to place value on being distinct from others and having a high status. People who endorse more of the HC items value interdependence, sociability, shared aims and goals. In contrast, people who endorse vertical aspects of collectivism strongly emphasize the importance of in-group integrity even at the cost of their own interests. In this study, we used the 16 items that have previously been found to have the highest factor loadings. For each of the 16 items, four for each subscale, ratings were made on a 7-point scale ranging from 1 indicating ‘strongly disagree’ to 7 indicating ‘strongly agree’. Therefore, the possible range of scores was 4 to 28 on each subscale. This questionnaire was translated and validated in the Arabic by one of us (SS) as part of her PhD research. The translated scale was reliable, with Cronbach’s alpha at acceptable levels for each subscale (HI = .852, VI = .674, HV = .746, VC = .803), and principal components analysis demonstrated the expected four-factor structure.

#### Perception of control

General sense of control was measured using the Sense of Control Measure [27] that was translated and validated in the pilot study reported above. This comprises four items measuring perceived mastery (e.g., ‘I can do just about anything I put my mind to’) and eight items measuring perceived constraints (e.g., ‘Other people determine most of what I can and cannot do’). Each item is scored 1 to 7, strongly disagree to strongly agree, with perceived constraints items being reverse scored. Scores can range on mastery from 7 to 28, and on constraints from 8 to 56, with higher values representing higher levels of perceived control. In this study, Cronbach’s alpha gave .592, .728, and .694 respectively for mastery, constraint and perceived control, which is at the level of acceptability.

### Procedure

Ethical approval for conducting this study was obtained from King Abdulaziz University in Saudi Arabia, and all measures were prepared for online administration using Google forum (online survey). The link for the online survey was distributed through social media (e.g. Whatsapp, telegram), text messages, and emails, to ensure that the sample included a wide variety of people of different ages (18 and above), educational levels, jobs, and interests, including Saudis and Arabic residents. It was made clear that participation was voluntary, and all participants gave their consent prior to their participation. Participants who wished to stop participating at any time for any reason, were instructed that they could do so by simply closing the web page.

### Analysis

Mediation analyses were tested using non-parametric bootstrapping procedures in order to re/sample the distribution of indirect effects. This procedure uses 5000 re-samples in order to calculate the 95% confidence limits for direct and indirect effects (using PROCESS for SPSS [37, 38]). Moderation hypotheses were tested by calculating interaction terms (i.e. perception of control × values), and entering each variable individually, followed by the interaction terms, into a block-wise linear regression. First, however, the interaction terms were transformed to ensure that the multicollinearity assumption was not violated. Our previous work shows that a sample size of approximately 500 participants is appropriate to test these hypotheses [39], thus the achieved sample of 614 participants is more than adequate.

## Results

Participants completed measures of perceived control, cultural values, and mood disorder symptoms (depression and bipolar symptoms). In order to test our hypotheses, we carried out a series of mediation and moderation analyses, including perceived control first as an overarching concept, and then with perceived mastery and constraints as distinct components of perceived control. We tested the models with the symptoms of depression and then bipolar disorder as outcome variables.

### Mediation hypotheses

These hypotheses were tested with endorsed cultural values entered into the models simultaneously for each symptom set.

#### Depression symptoms

**Fig 2:**
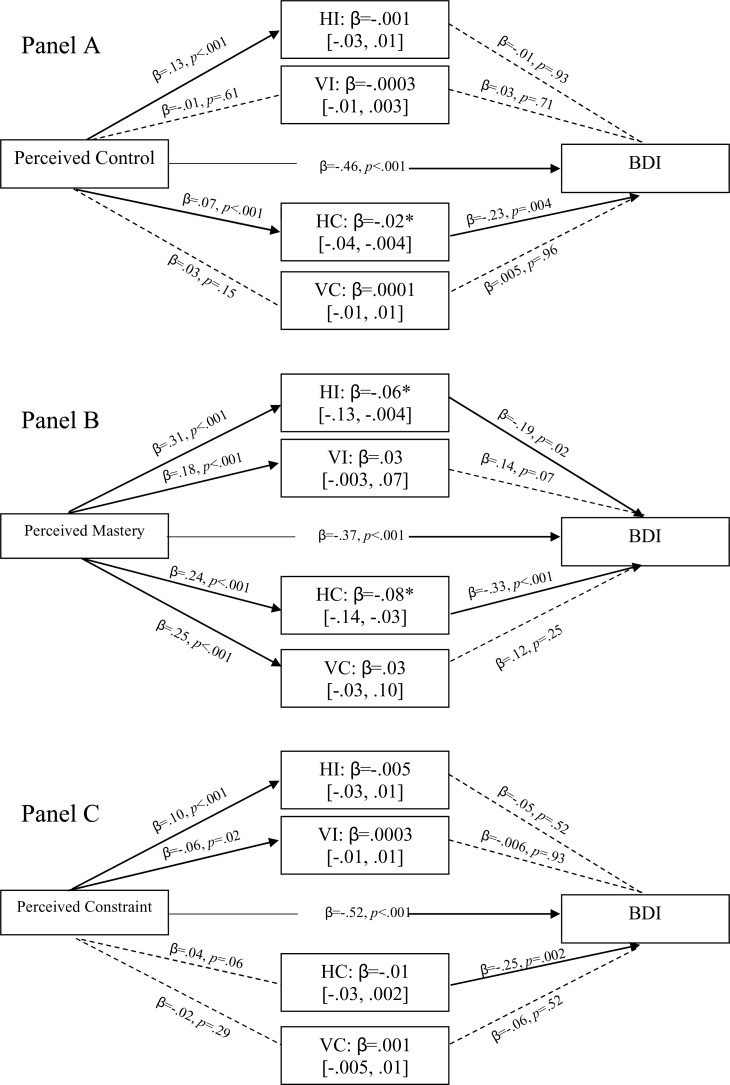
**Direct and indirect relationships between perceived control (A), and its components, Perceived Mastery (B) and Perceived Constraints (B), and BDI scores, through pathways indicating endorsed cultural.** NB: HI = Horizontal Individualism; VI = Vertical Individualism; HC = Horizontal Collectivism; VC = Vertical Collectivism.—Dotted lines represent non-significant pathways; Solid lines represent significant pathways.

Fig 2 shows the direct and indirect relationships between perceived control (panel A), perceived mastery (B), perceived constraints (C) and BDI scores. Note that in the case of mastery and constraint scores, higher values represent higher levels of perceived control due to reverse scoring on perceived constraint items. Fig 2.A shows that higher levels of perceived control predict lower levels of depression and vice versa, *β* = -.46, *p* < .001, horizontal collectivism adds to this effect significantly because higher levels of perceived control predict higher level so HC, and lower levels of Depression *β* = -.02 [-.04, -.004].

Partitioning perceived control into its constituent parts shows that perceived mastery is entirely responsible for the pattern in relation to cultural values. Rather than perceived control in general, it is perceived mastery that is important in relation to cultural values. Thus, high levels of mastery predict high levels of HC and HI which predicts low levels of depression, HC: β = -.08 [-.14, -.03]; HI: β = -.06 [-.13, -.004]. In contrast, a high sense of control in relation to constraint (i.e. low perceived constraint) directly predicts lower depressed mood, *β* = -.52, *p* < .001.

#### Bipolar symptoms

**Fig 3:**
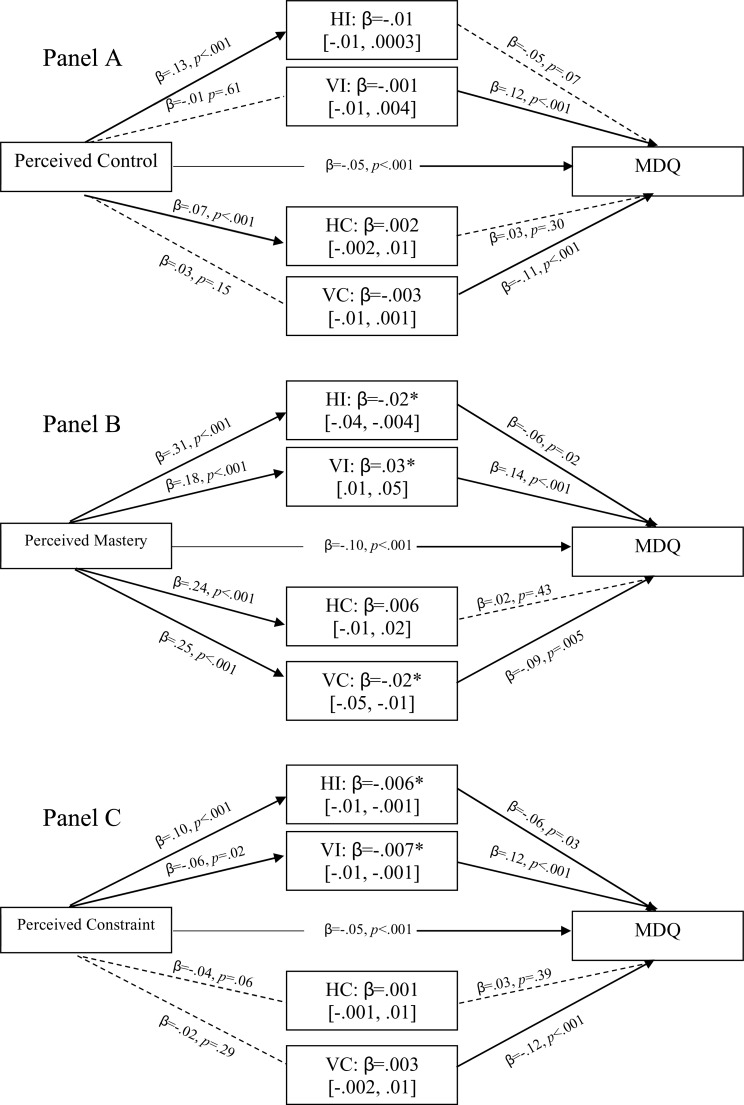
Direct and indirect relationships between perceived control (A), and its components, Perceived Mastery (B) and Perceived Constraints (B), and MDQ scores, through pathways indicating endorsed cultural. NB: HI = Horizontal Individualism; VI = Vertical Individualism; HC = Horizontal Collectivism; VC = Vertical Collectivism.—Dotted lines represent non-significant pathways; Solid lines represent significant pathways.

Fig 3 shows the direct and indirect relationships between perceived control (panel 3.A), perceived mastery (3.B), perceived constraints (3.C) and MDQ scores. For perceived control as an overarching concept, the picture is straightforward, higher levels of perceived control predict lower MDQ scores, *β* = -.05, *p* < .001, with no mediation by cultural values. However, Fig 3B and 3C show a more complex picture. In the case of mastery 3.B, the direct relation is stronger, β = -.10, p < .001, and is mediated by HI, β = -.02 [-.04, -.004], VI, β = .03 [.01, .05], and VC, *β* = -.02 [-.05, -.01]. In the case of HI and VC, this mediated relationship is negative meaning that high HI and VC, along with perceived control, predict lower levels of symptoms. VI on the other hand is a positive mediator, such that high levels of mastery predict high levels of VI and high levels of bipolar symptoms. In the case of constraints, Fig 3C. individualism is the only mediator, with HI, β = -.006 [-.01, -.001], and VI, β = -.007* [-.01, -.001], giving significant pathways from high perceived constraint to low symptoms.

### Moderation hypotheses

The moderator variables (interaction terms) were entered simultaneously into the models for symptom set. In order to avoid repetition and because the direct relationships between variables are identical to the mediation analyses, we have summarised these results, with the full analysis given in S1 Fig.

#### Depression symptoms

None of the interaction terms had a significant effect on the relationship between perceived control, or its constituents, and BDI scores (See S1 Fig).

#### Bipolar disorder symptoms

There were no significant interaction terms (see S2 Fig), except for the perceived constraints model. HI was a significant moderator of the perceived constraints to MDQ relationship, β = -.01* [-.01, -.001]. Follow up analyses, based on non-transformed data showed that the PC MDQ relationship was stronger for people who endorsed fewer HI values. For people in the low HI category, higher perceived constraints strongly predicted higher MDQ scores: HI_Low_, *r* = .67**. However, for medium, moderate and high HI scores ranging from 6 to 28, the relation was negative such that higher perceived constraints predicted lower MDQ scores, but this relationship was weaker, *r*_max_ = -.439*.

## Discussion

In this study, we set out to investigate whether the relationships between perception of control and mood disorder symptoms were mediated and / or moderated by the cultural values endorsed by these participants who were recruited from a largely collectivist society. First, however, we translated and validated a frequently used measure of perception of control (Sense of control: [27]) into the Arabic language. This is the first contribution of this paper. The subsequent theoretical study delivered further contributions and showed that, in all cases, the direct relation from perceived control was negative, such that higher levels of control were linked to lower symptom levels. However, culture did influence the relation between control and bipolar symptoms levels. Specifically, for the mediation model, perceived mastery was most frequently implicated. Data were generally consistent the idea of a boost to the protective effect of perceived control, with lower symptoms levels observed. There was only one exception to this pattern. High levels of perceived mastery predicted higher endorsed vertical individualistic values which further predicted higher levels of bipolar symptoms. This latter finding is consistent with previous findings involving behavioural measures of perceived control and a European sample [24]. There was less evidence for moderation effects, perhaps unsurprisingly as this was a monocultural sample. Moderation was limited to perceived constraints and bipolar symptoms, such that for participants who did not endorse and disagreed or strongly disagreed with horizontal individualist values, higher levels of constraints were correlated with higher symptom levels. However, for those who were either ambivalent or who agreed with the same values, higher constraints were correlated with low symptom levels. In the rest of the discussion, we discuss these findings in more detail as well as their theoretical and practical implications.

In this Saudi sample, high levels of control directly predicted lower levels of depression and bipolar symptoms. This direct relation was strong, and evident for general perception of control and both of its components, mastery and constraints. Thus, these findings lend support to the traditional model. However, the current findings do not speak to cross-cultural contrasts reported in other studies showing weaker relations in collectivist samples [22]. Here, however, effect sizes for depression were large, albeit smaller for bipolar symptoms. The current data are consistent with Steptoe et al.,’s analysis [23] which showed no attenuation of effect sizes across cultures and suggests that the impact of perception of control on mental health is universal.

However, before continuing this discussion and, as a note of caution, we should note that there is considerable variation in the manner in which control is measured across studies [40]. For example, single item survey measures [22], validated instruments [23] and behavioural measures [24] have been used. In addition, numerous constructs have been related to perceived control with terms such as locus of control and perceived control often used synonymously [40]. As an example, Cheng [28] reported that the relation between control and anxiety was weaker in collectivist samples, though the relation with depression symptoms did not differ. Interestingly, that particular meta-analysis involved locus of control measures (for a description see [41]), which describe the control ‘orientation’ of individuals and their beliefs that events are controlled internally (self-factors) or externally, as opposed to measuring the extent of perceived control, and might explain some of the differences between the sets of findings. In the current study, we were not only keen to look at control as a general construct, but we also wanted to examine the components of mastery and constraint, in order to better understand how culture intervenes in PCMHM.

Therefore, we tested two types of models. The first test was of a process-based account, a values-based causal pathway to mood disorder symptoms. Perceived control is considered to be a generalised belief based on fundamental learning processes, which influences values that are assumed to be situationally based and not fixed, which in turn influences mood state. The second model tested is a more conventional individual differences moderator model, which assumes that the strength and direction of the control-symptom relation will be affected by the values which a person endorses.

The mediator results showed that perceived mastery was related to cultural values on all four dimensions (VI, HI, VC, HC), and this relation was positive. Perceived mastery refers to a person’s perception of their own competence, that their behavior is effective [40], and that they can ‘do’ the behavior they want to engage in (“I can do just about anything I set my mind to”) [27]. So, a person in this sample who rated their own competence highly was also more likely to endorse items on self-reliance, to value winning, as well as valuing coworkers and family–a generally positive set of values. These results also showed that the pathway from perceived mastery to mood disturbance was most frequently mediated by cultural values in comparison to perceived constraints and general sense of control. For the most part, the results were consistent with the idea that the endorsement of the values mentioned above enhanced the pathway from perceived control to symptoms, in that they were associated with lower levels of mood disturbance symptoms.

There was one exception to this pattern. In the pathway from mastery to bipolar symptoms, results suggested that high levels of vertical individualism diminished the protective effect of perceived control. This finding is consistent with our previous work carried out with European samples [24]. In those studies, high levels of perceived control and individualist values (VI) were consistently associated with increased euphoric symptoms of bipolar disorder. The measures of control used in that study were experimental with higher levels of perceived control being consistent with illusory control, as participants rated a situation in which they had no control. This suggests that the impact of vertical individualism on bipolar symptoms is consistent across behavioural and psychometric measures, as well as across cultures. Clearly, however, given that the present study involves cross-sectional correlational data, they cannot be taken as strong evidence for a causal pathway model.

In spite of this an interesting aspect of these findings supports the causal process version of the PCMHM because perceived control was associated with the extent to which people endorsed particular cultural values. In other words, situational factors, such as the control that one perceives, influence the endorsement of values. This is not a new idea. For example, Triandis [42] argued that people are capable of using different patterns of responding depending on the situation they find themselves in. One example given is of group level interaction such as when the “…ingroup is under attack from outsiders” ([42] p. 411). The current evidence is consistent with the idea that an individual difference factor, such as perceived control, can also influence a person’s values profile, and have a significant effect on their mood state. We do note again, however, that whilst mediation models are based on a theoretical causal hypothesis, it is not possible to provide strong evidence for the causal pathway using a correlational design. Thus, any causal pathway conclusions should be treated with appropriate caution.

As noted above, this study did not aim to test cross cultural comparisons, although we did test the individual difference, moderator version of the PCMHM. Within this collectivist sample, whilst there was enough variability in the endorsement of values such that there was evidence of indirect effects, there was, perhaps unsurprisingly, less evidence of endorsed values being a moderating factor in terms of the control-symptom relationship. This may suggest that the population sampled was relatively homogenous, in relation for example, to a more multi-cultural society in which there are distinct values-linked subgroups present. This is because moderation is essentially an analysis of subgroups within a population. There was one exception to this where, for those disagreed with disagreed with HI values, high constraints strongly predicted higher bipolar symptoms. Agreement with HI values produced the opposite though weaker relationship. HI values are around a person valuing their own distinctive identity, without wanting status [36]. Thus, high symptom levels are predicted when a person disagrees with the value of that distinctive identity and feels that there are constraints on their ability to be in control. It is interesting to note that the mediator and moderator models produced distinct sets of findings, with perceived mastery model being mediated by VI and the perceived constraints model being moderated by HI. These findings link to our rationale for testing both models and are consistent with the idea that the models are not mutually exclusive. We do note, however, that mediation and moderation aspects of the models are relatively weak in comparison strong direct relationships between perceived control and symptoms.

Another note of caution is that testing control perceptions as the starting point of the model is not the only possibility. Control perceptions could be a cause of altered mood or a consequence, or both, and we do not claim to distinguish between these possibilities. It could be argued, however, is that perceived control is one of the fundamentals of human functioning and is driven by basic learning processes [8, 43]. Similarly, Skinner [25] argues that the consequences of perception of control are cognitive, and that the sense of having control allows people to retain access to higher order cognitive capacities. Thus, we argue here and report evidence elsewhere [29] that the PCMHM can function as a causal pathway model. That being said, this does not exclude or invalidate the moderator model. As tested in cross cultural studies, there are differences between groups of similar individuals in the control-symptom relationship. Here we observed this effect in constraint-control relationship. This further supports the construct of perceived control, as composed of distinct components [31], and emphasises the importance of testing these.

## Conclusions

The current study adds to the literature on the links between perceived control and cultural values. The findings here show that, in a sample derived from a collectivist culture, higher levels of perceived control are generally consistent with lower levels of mood disturbance, and for the most part, similar values seem to enhance this trend. This suggests that therapists using traditional CBT formulations in a collectivist setting can be confident of the applicability of this approach. Specifically, however, in the case of clients with bipolar disorder, therapists should be aware that cultural values linked to perceived control could influence symptoms, though of course further research is needed. Taken together, the current findings suggest that cultural values that are consistent with the positive effects of perceived control tend to be linked to stronger positive effects of perceived control on mood disturbance. Collectivist values do not seem to interfere with any mood enhancing effects of perceived control. These data should reassure those who have had concerns about the applicability of individualistic theories to collectivist client groups.

## Supporting information

S1 Fig The relationship between perceived control and Beck Depression Inventory (BDI) Scores. NB. Significant pathways are indicated by solid lines. 95% confidence limits are given in brackets.
(EPS)

S2 Fig The relationship between perceived control and MDQ Scores. NB. Significant pathways are indicated by solid lines. 95% confidence limits are given in brackets.
(EPS)

## References

[1] Taylor SE, Brown JD. Illusion and well-being—a social psychological perspective on mental-health. PsyB. 1988;103(2):193–210. ISI:A1988M391400004. 3283814

[2] Ell KO, Mantell JE, Hamovitch MB, Nishimoto RH. Social support, sense of control, and coping among patients with breast, lung, or colorectal cancer. Journal of Psychosocial Oncology. 1989;7(3):63–89.

[3] Macrodimitris SD, Endler NS. Coping, control, and adjustment in Type 2 diabetes. Health Psychol. 2001;20(3):208 11403218

[4] Bosma H, Marmot MG, Hemingway H, Nicholson AC, Brunner E, Stansfeld SA. Low job control and risk of coronary heart disease in Whitehall II (prospective cohort) study. Bmj. 1997;314(7080):558 10.1136/bmj.314.7080.558 9055714 PMC2126031

[5] Geers AL, Rose JP, Fowler SL, Rasinski HM, Brown JA, Helfer SG. Why does choice enhance treatment effectiveness? Using placebo treatments to demonstrate the role of personal control. JPSP. 2013;105(4):549. 10.1037/a0034005 23915042

[6] Lundberg J, Bobak M, Malyutina S, Kristenson M, Pikhart H. Adverse health effects of low levels of perceived control in Swedish and Russian community samples. BMC Public Health. 2007;7(1):314 10.1186/1471-2458-7-314 17980033 PMC2200648

[7] Infurna FJ, Gerstorf D, Ram N, Schupp J, Wagner G. Long-term antecedents and outcomes of perceived control. Psychology and Aging. 2011;26(3):559 10.1037/a0022890 21517184 PMC3319760

[8] Alloy LB, Abramson LY. Judgement of contingency in depressed and non-depressed students: Sadder but wiser? Journal of Experimental Psychology-General. 1979;108(4):441–85. 528910 10.1037//0096-3445.108.4.441

[9] Seligman ME. Helplessness: On development, depression and death. New York: W. H. Freeman and Company; 1975.

[10] Pagnini F, Bercovitz K, Langer E. Perceived control and mindfulness: Implications for clinical practice. Journal of Psychotherapy Integration. 2016;26(2):91.

[11] Msetfi RM, Cavus HA, Brosnan L. Enhanced Attention to Context Increases Perceived Control in Mild Depression. Quarterly Journal of Experimental Psychology. 2016;69(6):1073–81. 10.1080/17470218.2016.1138134 26822778

[12] Msetfi R, O'Sullivan D, Walsh A, Nelson J, Van de Ven P. Using Mobile Phones to Examine and Enhance Perceptions of Control in Mildly Depressed and Nondepressed Volunteers: Intervention Study. Jouranl of Medical Internet Research mHealth uHealth. 2018;6(11). 10.2196/10114 PMC6251979 30413398

[13] Matute H. Detecting response-outcome independence in analytic but not in naturalistic conditions. Psychol Sci. 1996;7(5):289–93. 10.1111/j.1467-9280.1996.tb00376.x 1996-06306-006. First Author & Affiliation: Matute, Helena.

[14] Byrom N, Msetfi R, Murphy R. Two pathways to causal control: use and availability of information in the environment in people with and without signs of depression. Acta psychologica. 2015;157:1–12. 10.1016/j.actpsy.2015.02.004 25703605

[15] Yarritu I, Matute H, Vadillo MA. Illusion of control: the role of personal involvement. Experimental Psychology. 2014;61(1):38 10.1027/1618-3169/a000225 23948387 PMC4013923

[16] Matute H, Yarritu I, Vadillo MA. Illusions of causality at the heart of pseudoscience. BJP. 2011;102(3):392–405. 10.1348/000712610X532210 21751996

[17] Dixon MR, Hayes LJ, Ebbs RE. Engaging in 'illusory control' during repeated risk-taking. Psychological Reports. 1998;83(3, Pt 1):959–62. 10.2466/pr0.83.7.959-962 1999-00342-037. First Author & Affiliation: Dixon, Mark R. 9923175

[18] Moritz S, Thompson SC, Andreou C. Illusory Control in Schizophrenia. Journal of Experimental Psychopathology. 2014;5(2):113–22. 10.5127/jep.036113

[19] Balzan RP, Delfabbro PH, Galletly CA, Woodward TS. Illusory Correlations and Control Across the Psychosis Continuum: The Contribution of Hypersalient Evidence-Hypothesis Matches. 2013;201(4):319–27. 10.1097/NMD.0b013e318288e229 00005053-201304000-00009. 23538977

[20] Reuven-Magril O, Dar R, Liberman N. Illusion of control and behavioral control attempts in obsessive-compulsive disorder. JAP. 2008;117(2):334. 10.1037/0021-843X.117.2.334 18489209

[21] Markus HR, Kitayama S. Culture and the self: Implications for cognition, emotion, and motivation. PsychologR. 1991;98(2):224–53. 10.1037/0033-295x.98.2.224 1991-23978-001. First Author & Affiliation: Markus, Hazel R.

[22] Sastry J, Ross CE. Asian ethnicity and the sense of personal control. SPsy. 1998;61(2):101–20. 10.2307/2787064 WOS:000074678300003.

[23] Steptoe A, Tsuda A, Tanaka Y, Wardle J. Depressive symptoms, socio-economic background, sense of control, and cultural factors in university students from 23 countries. International Journal of Behavioral Medicine. 2007;14(2):97–107. 10.1007/bf03004175 WOS:000247841100007. 17926438

[24] Msetfi RM, Kornbrot DE, Matute H, Murphy RA. The relationship between mood state and perceived control in contingency learning: effects of individualist and collectivist values. Frontiers in psychology. 2015;6. 10.3389/fpsyg.2015.01430 PMC4586436 26483707

[25] Skinner EA. Seven Guideposts to the Study of Perceived Control across the Lifespan In: Reich JW, Infurna FJ, editors. Perceived control: Theory, research and practice in the first 50 years: Oxford University Press; 2015.

[26] Dobson K, Franche RL. A conceptual and empirical review of the depressive realism hypothesis. Can J Behav Sci-Rev Can Sci Comport. 1989;21(4):418–33. ISI:A1989AX14000007.

[27] Lachman ME, Weaver SL. The sense of control as a moderator of social class differences in health and well-being. JPSP. 1998;74(3):763. 10.1037//0022-3514.74.3.763 9523418

[28] Cheng C, Cheung S-f, Chio JH-m, Chan M-pS. Cultural meaning of perceived control: A meta-analysis of locus of control and psychological symptoms across 18 cultural regions. PsyB. 2013;139(1):152–88. 10.1037/a0028596 2012-13975-001. First Author & Affiliation: Cheng, Cecilia. 22642229

[29] Brislin RW. Back-translation for cross-cultural research. Journal of cross-cultural psychology. 1970;1(3):185–216.

[30] Senan S. Developmental factors and psychological processes underlying vulnerability to bipolar disorder across individualistic and collectivistic cultures: University of Limerick; 2014.

[31] Beck AT, Ward CH, Mendelson M, Mock J, Erbaugh J. An inventory for measuring depression. Archives of General Psychiatry. 1961;4:561–71. 10.1001/archpsyc.1961.01710120031004 13688369

[32] Bumberry W, Oliver JM, McClure JN. Validation of the Beck Depression Inventory in a university population using psychiatric estimate as the criterion. Journal of Consulting and Clinical Psychology. 1978;46(1):150–5.

[33] Abdel-Khalek A. Internal consistency of an Arabic Adaptation of the Beck Depression Inventory in four Arab countries. Psychological reports. 1998;82:264–6. 10.2466/pr0.1998.82.1.264 9520563

[34] Hirschfeld RMA, Williams JBW, Spitzer RL, Calabrese JR, Flynn L, Keck PE Jr., et al Development and validation of a screening instrument for bipolar spectrum disorder: The Mood Disorder Questionnaire. The American Journal of Psychiatry. 2000;157(11):1873–5. 10.1176/appi.ajp.157.11.1873 2000-16354-020. First Author & Affiliation: Hirschfeld, Robert M. A. 11058490

[35] Dodd AL, Mansell W, Sadhnani V, Morrison AP, Tai S. Principal components analysis of the Hypomanic Attitudes and Positive Predictions Inventory and associations with measures of personality, cognitive style and analogue symptoms in a student sample. Behavioural and Cognitive Psychotherapy. 2010;38(01):15–33. 19857364 10.1017/S1352465809990476

[36] Triandis HC, Gelfand MJ. Converging measurement of horizontal and vertical individualism and collectivism. JPSP. 1998;74(1):118–28. 10.1037/0022-3514.74.1.118 1997-38342-009. First Author & Affiliation: Triandis, Harry C.

[37] Hayes AF. PROCESS: A versatile computational tool for observed variable mediation, moderation, and conditional process modeling. Manuscript submitted for publication. 2012.

[38] Hayes AF. Beyond Baron and Kenny: Statistical mediation analysis in the new millennium. ComM. 2009;76(4):408–20. 10.1080/03637750903310360 2009-23463-004. First Author & Affiliation: Hayes, Andrew F.

[39] Msetfi RM, Jay S, O’Donnell AT, Kearns M, Kinsella EL, McMahon J, et al Restricted reproductive rights and risky sexual behaviour: How political disenfranchisement relates to women’s sense of control, well-being and sexual health. Journal of Health Psychology. 2017;0(0):1359105317736784 10.1177/1359105317736784 . 29076402

[40] Skinner EA. A guide to constructs of control. JPSP. 1996;71(3):549–70. 10.1037/0022-3514.71.3.549 1996-01799-010. 8831161

[41] Rotter JB. Generalized expectancies for internal versus external control of reinforcement. Psychological Monographs: General and Applied. 1966;80(1):1. 5340840

[42] Triandis HC. The psychological measurement of cultural syndromes. AmP. 1996;51(4):407–15.

[43] Msetfi RM, Murphy RA, Simpson J, Kornbrot DE. Depressive realism and outcome density bias in contingency judgements: The effect of context and the inter-trial interval. Journal of Experimental Psychology: General. 2005;134(1):10–22. 15702960 10.1037/0096-3445.134.1.10

